# Psychosocial outcomes of preschool children with a history of preterm birth: a cross-sectional observational study

**DOI:** 10.3389/fped.2026.1834826

**Published:** 2026-07-23

**Authors:** Hongli Sun, Jia Wang, Huan Qiang

**Affiliations:** 1 Department of Sociology, School of Humanities and Social Sciences, Xi'an Jiaotong University, Xi'an, Shaanxi, China; 2 Shaanxi Institute for Pediatric Diseases, Xi'an Key Laboratory of Children's Health and Diseases, Xi'an Children's Hospital, Affiliated Children's Hospital of Xi'an Jiaotong University, Xi'an, Shaanxi, China; 3 School of Politics, Law & Public Administration, Yan'an University, Yan'an, Shaanxi, China; 4 Department of Child Health Care, Xi'an Children's Hospital, Affiliated Children's Hospital of Xi'an Jiaotong University, National Regional Children's Medical Center (Northwest), Xi'an, Shaanxi, China

**Keywords:** internalizing problems, preschool children, preterm birth, psychosocial outcomes, strengths and difficulties questionnaire

## Abstract

**Background:**

Preterm birth is associated with long-term neurodevelopmental challenges, yet evidence on psychosocial outcomes in Chinese preschool children, particularly in less developed regions, remains scarce.

**Methods:**

A cross-sectional study was conducted in a western Chinese city from February 28 to March 5, 2025. Using stratified cluster sampling, 20,913 parent–child dyads were recruited from 189 kindergartens. Preterm birth (< 37 weeks gestation) was parent-reported. For the assessment of psychosocial functioning, the Chinese adaptation of the Strengths and Difficulties Questionnaire (SDQ) was utilized, with its focus on total difficulties, internalizing problems, externalizing problems and prosocial behavior. Multivariable regression models were used, adjusting for child age and gender, child birth weight, number of children, parental age, parental gender, education level, employment status, marital status, annual family income, smoking status, alcohol intake status, and parental depressive symptoms (CES-D score). Subgroup analyses explored effect modification.

**Results:**

Of 20,913 children (mean age 4.82 ± 0.89 years), 860 (4.1%) were preterm. After full adjustment, preterm birth was significantly associated with increased odds of total difficulties (OR = 1.32, *p* = 0.0015), but not with internalizing problems (OR = 1.07, *p* = 0.4153), externalizing problems (OR = 1.02, *p* = 0.8083), or prosocial behavior (OR = 0.89, *p* = 0.1009).

**Conclusions:**

Preterm birth in preschool children from Western China was associated with higher odds of total difficulties, but not with internalizing problems, externalizing problems, or prosocial behavior after full adjustment. Stratified interaction analyses revealed that these associations differed by alcohol intake status (for total difficulties) and parental education level (for prosocial behavior) after FDR correction.

## Introduction

Preterm birth, which is defined as delivery occurring prior to 37 weeks of gestation, constitutes a critical public health issue worldwide (1, 2). Substantial advances in perinatal medicine and neonatal intensive care have significantly improved the survival rate of preterm infants, thereby shifting research priorities from immediate postnatal survival to long-term neurodevelopmental and psychosocial outcomes. Preterm birth is increasingly recognized as a key factor associated with impaired psychosocial development in children (3, 4). Early childhood is a period of high neural plasticit, during which early adverse physiological experiences and environmental factors may influence neurodevelopmental pathways, profoundly affecting psychosocial outcomes and long-term adaptive functioning (5). This has implications not only for immediate social integration but also for future academic achievement and overall mental health outcomes.

Although the negative impact of preterm birth on psychosocial development is well-documented (6–8), its specific manifestations and influencing factors require further investigation. Current evidence suggests that preterm children may exhibit behavioral difficulties in childhood (9–12) A longitudinal study of 296 Australian children found that those born moderate-to-late preterm (32–36 weeks) had significant neurodevelopmental challenges at age 9 compared to those born ≥37 weeks (13). These included lower full-scale IQ, poorer academic performance in mathematics and reading, and more behavioral difficulties (13). Early developmental delay at age 2 was a strong predictor of later school-age impairments, suggesting that early screening can help identify at-risk children (13). Children born extremely preterm (<28 weeks) face high risks of neurodevelopmental impairments (cognitive, motor, behavioral), affecting school performance and health (14). However, preterm children also face significant socio-emotional challenges, including reduced accuracy in recognizing positive facial emotions and poorer social and behavioral outcomes compared to full-term peers. Early difficulties in emotion recognition correlate with later socio-emotional functioning (15). Studies also indicate altered stress responses in preterm infants, characterized by muted emotional reactivity and uncoupling of cortisol and behavioral responses (16). These deficits often persist into school age, with many preterm children showing impaired emotion recognition and social cognition, potentially linked to socioeconomic and environmental factors. Early identification and support are crucial (15, 17, 18). Furthermore, preterm children face unique social interaction challenges, with a significantly elevated risk of autism spectrum disorder (ASD) and potentially distinct clinical features compared to non-preterm children with ASD (19, 20). However, existing studies often report inconsistent results and are predominantly based on Western populations. Given the substantial influence of culture, region, and healthcare systems on psychosocial development, large-scale, representative epidemiological studies on preterm preschoolers in China—particularly in less developed western urban regions—remain scarce. This study was conducted in a major urban center in Western China to provide region-specific evidence from an area with evolving healthcare resources.

To fill this research gap, we carried out a large-scale cross-sectional study in a city located in Western China, aiming to explore the association between preterm birth and psychosocial outcomes among preschool-aged children. Using a stratified cluster sampling approach, 20,913 parent–child dyads were recruited from 189 kindergartens. Children's psychosocial functioning was evaluated via the parent-reported Strengths and Difficulties Questionnaire (SDQ), which encompasses four dimensions: total difficulties, internalizing problems, externalizing problems, and prosocial behavior (20, 21). Multivariable logistic regression models were employed to analyze the independent association between preterm birth and various psychosocial outcomes, adjusting for potential confounders such as parental and child demographic characteristics, family socioeconomic status, and parental health behaviors. Stratified interaction analyses were conducted to explore whether key sociodemographic and behavioral factors showed statistical interactions with these associations (i.e., effect modification on the multiplicative scale), assessed via interaction terms in multivariable logistic regression models.

## Methods

### Participants and data collection

This study was conducted in collaboration with the Education Bureau and local educational institutions in a city in Western China. To obtain a representative sample of kindergarten-aged children, a stratified cluster sampling approach was adopted. The sampling procedure involved two key stages: first, the city was divided into 13 administrative districts. Public kindergartens were then randomly selected from each stratum based on the official kindergarten registry obtained from the local Education Bureau. The sample size for each stratum was determined by proportional allocation according to the relative population of children in each district. A total of 189 kindergartens participated as study sites, and all children and their guardians were invited to take part.

Data collection was conducted from February 28 to March 5, 2025, and targeted preschool children aged 3–6 years attending junior, middle, and senior kindergarten classes. Guardians provided informed consent prior to their participation in the study, and relevant data were collected through self-administered questionnaires filled out by the guardians. The initial dataset included 25,017 parent–child dyads. To ensure data quality and sample representativeness, a structured data cleaning process was applied with the following sequential exclusions: dyads with non-parent caregivers (*n* = 2,408) and records with implausible or missing values in variables (e.g., age and child birth weight) (*n* = 1696). The final analytic sample comprised 20,913 parent–child dyads that fulfilled the core inclusion criteria—questionnaires completed by one of the child's parents within the designated preschool age range—thus supporting the internal validity of subsequent analyses pertaining to parental reports and early childhood developmental outcomes.

### Exposure variable

The primary exposure variable in this study was the birth status of the child, measured through a questionnaire item posed to guardians: “What is the birth status of your child (in this survey)?” The response options were dichotomous, allowing guardians to classify their child's birth as either “non-preterm” or “preterm.” For the purpose of data analysis, full-term birth was defined as a gestational age of 37 weeks or more, whereas preterm birth was specified as a gestational age of fewer than 37 weeks. Detailed gestational age in weeks was not available in our dataset, as preterm status was collected via a parent-reported dichotomous question. Therefore, classification of prematurity severity (extremely, very, moderate-to-late preterm) was not possible.

### Outcome variable

The mental health status of children was assessed using the globally validated Strengths and Difficulties Questionnaire (SDQ), which has established reliability and validity in Chinese children aged 3–17 years (22). Data were collected using the parent-reported version of the SDQ, consisting of 25 items rated on a 3-point Likert scale (0 = not true, 1 = somewhat true, 2 = certainly true). The items are grouped into five subscales: Emotional Symptoms, Conduct Problems, Hyperactivity/Inattention, Peer Relationship Problems, and Prosocial Behavior.

Internal consistency reliability of the SDQ subscales was assessed using Cronbach's alpha coefficient. Since the SDQ employs a 1–3 response format (1 = Not True, 3 = Certainly True) with reverse-scored items (Items 7, 11, 14, 21, and 25), all reverse-scored items were recoded prior to analysis (1→3, 2→2, 3→1). The alpha function from the R package psych was used with the check.keys = TRUE option to automatically detect and correct any remaining directional inconsistencies. Item-level diagnostic analyses were conducted to examine the corrected item-total correlations (r.cor). Two items demonstrated weak associations with their respective subscale constructs: Item 7 (“Generally obedient, usually does what adults request”) in the Conduct Problems subscale (r.co*r* = 0.135) and Item 23 (“Prefers to be with adults rather than with other children”) in the Peer Relationship Problems subscale (r.co*r* = 0.065). These items were removed to improve subscale reliability, following recommendations in the literature (23, 24).

For analysis, a total difficulties score was calculated by summing the scores of the four subscales: Emotional Symptoms, Conduct Problems, Hyperactivity/Inattention, and Peer Relationship Problems. Based on Chinese norm-referenced cut-off standards, the total score was dichotomized into “normal” (0–14) and “at-risk” (>14, including borderline and abnormal ranges). The following subscale-specific cut-offs were applied to identify at-risk status: > 3 for Emotional Symptoms, > 2 for Conduct Problems, > 6 for Hyperactivity/Inattention, > 4 for Peer Problems, and < 6 for Prosocial Behavior deficits. Children were classified as being at risk (yes/no) for internalizing problems based on emotional symptoms, and for externalizing problems based on conduct problems or hyperactivity/inattention (22, 25). In the analyses, all SDQ subscales were treated as categorical variables to examine their associations with the exposure factors.

### Covariates

Based on previous research findings and clinical expertise, covariates were chosen for this study (18). The following factors were incorporated as covariates: (1) Categorical variables: parental gender, child gender, smoking status, alcohol intake status, education level, annual family income, employment status, marital status, number of children; (2) Continuous variables: parental age, child age, child birth weight, parental depressive symptoms (CES-D score). Variables were categorized as follows: child age (< 4, 4–5, ≥ 5 years); child gender (boys, girls); child birth weight (< 2,500 g or > 4,000 g, ≥ 2,500 to 4,000 g); number of children (1, 2, ≥ 3); parental age (< 30, 30–45, ≥ 45 years); parental gender (men, women); education level (≤ junior high school, high school/junior college, ≥ undergraduate); annual family income (< 30, 30–100, ≥ 100 thousand yuan); employment status (working, not working); marital status (married/living with partner, others); smoking status (no, yes); alcohol intake status (no, yes); and CES-D (≥ 16, < 16).

### Statistical analysis

Data were analyzed using R (version 4.3.2) and Empower Stats (version 3.0). Participants were first stratified into two groups based on preterm birth status (non-preterm vs. preterm). Categorical variables are reported as frequencies and percentages, and continuous variables as means with standard deviations (SD). Between-group differences were evaluated using the *χ*^2^ test for categorical variables, Student's t-test for normally distributed continuous variables, and the Mann–Whitney U test for non-normally distributed continuous variables. To account for the potential clustering effect of kindergartens (intra-class correlation), we conducted sensitivity analyses using Generalized Estimating Equations (GEE) with an exchangeable correlation structure, with kindergarten as the clustering unit. Preliminary multilevel null models yielded negligible intraclass correlation coefficients (ICC < 0.01 for all outcomes; Supplementary Table S2), suggesting minimal kindergarten-level clustering. The GEE results were consistent with those from standard logistic regression (Supplementary Table S1), confirming the robustness of our findings. As shown in Table 3, the variance inflation factor (VIF) values are all below the commonly accepted threshold, indicating no significant multicollinearity among the variables.

The association between preterm birth and psychosocial outcomes was examined through multivariable logistic regression (20). Two models were fitted: a crude model without covariate adjustment and a fully adjusted model that accounted for child age and gender, child birth weight, number of children, parental age, parental gender, education level, employment status, marital status, annual family income, smoking status, alcohol intake status, and parental depressive symptoms (CES-D score). Stratified interaction analyses were conducted using multivariable-adjusted logistic regression. Each stratified interaction analyses was fully adjusted for all covariates except the stratification variable. Continuous covariates were categorized to facilitate stratification and clinical interpretation, based on the distribution of the study sample. Interaction terms were incorporated into the multivariable logistic regression models, and their significance was evaluated using likelihood ratio tests to explore potential effect modification by subgroup variables. A two-tailed *P* value < 0.05 was considered statistically significant. All stratified interaction analyses were exploratory, and False Discovery Rate (FDR) correction was applied to account for multiple comparisons (Supplementary Tables S5-S7). Significant interaction findings should be interpreted cautiously and require confirmation in future studies.

## Results

### Demographic and clinical characteristics

The demographic and clinical characteristics of the study population are summarized in Table 1. A total of 20,913 parent–child dyads were included, comprising 20,053 dyads of non-preterm children and 860 dyads of preterm children. The mean ages of parents and children were 34.76 ± 4.57 and 4.82 ± 0.89 years, respectively. Compared with the non-preterm group, the preterm group exhibited statistically significant differences in parental age (34.75 ± 4.56 vs. 35.09 ± 4.94 years), child age (4.82 ± 0.89 vs. 4.74 ± 0.88 years), child gender, annual family income, marital status, CES-D scores, total difficulties, internalizing problems, and prosocial behavior (all *p*-values < 0.05). No statistically significant differences were observed in parental gender, child birth weight, number of children, education level, employment status, smoking status, alcohol intake, or externalizing problems (all *p*-values ≥ 0.05).

**Table 1:** Demographic and clinical characteristics of the study population by preterm status.

Variables	Mean + SD/ N (%)	Non-preterm	Preterm	*P*-value
N	20,913	20,053 (95.9%)	860 (4.1%)	
Parental age (years)	34.76 ± 4.57	34.75 ± 4.56	35.09 ± 4.94	0.0447
Child age (years)	4.82 ± 0.89	4.82 ± 0.89	4.74 ± 0.88	0.0068
Parental gender				1
Men	5,003 (23.9%)	4,797 (23.9%)	206 (24%)	
Female	15,910 (76.1%)	15,256 (76.1%)	654 (76%)	
Child gender				0.0304
Boys	10,832 (51.8%)	10,355 (51.6%)	477 (55.5%)	
Girls	10,081 (48.2%)	9,698 (48.4%)	383 (44.5%)	
Child birth weight (g)	3,261 ± 482.16	3,261.57 ± 481.58	3,247.7 ± 495.67	0.4213
Number of children				0.0979
1	6,008 (28.7%)	5,755 (28.7%)	253 (29.4%)	
2	12,585 (60.2%)	12,091 (60.3%)	494 (57.4%)	
≥ 3	2,320 (11.1%)	2,207 (11%)	113 (13.1%)	
Education level				0.4138
≤ Junior high school	5,645 (27%)	5,396 (26.9%)	249 (29%)	
High school diploma and junior college	9,234 (44.2%)	8,863 (44.2%)	371 (43.1%)	
≥ Undergraduate degree	6,034 (28.9%)	5,794 (28.9%)	240 (27.9%)	
Employment status				0.6842
Working	15,312 (73.2%)	14,688 (73.2%)	624 (72.6%)	
Not working	5,601 (26.8%)	5,365 (26.8%)	236 (27.4%)	
Annual family income, in thousands, ¥				0.0049
< 30	8,432 (40.3%)	8,041 (40.1%)	391 (45.5%)	
≥ 30 to 100	8,438 (40.3%)	8,130 (40.5%)	308 (35.8%)	
≥ 100	4,043 (19.3%)	3,882 (19.4%)	161 (18.7%)	
Marital status				<0.001
Married/Living with partner	20,326 (97.2%)	19,512 (97.3%)	814 (94.7%)	
Others	587 (2.8%)	541 (2.7%)	46 (5.3%)	
Smoking status				1
No	17,807 (85.1%)	17,075 (85.1%)	732 (85.1%)	
Yes	3,106 (14.9%)	2,978 (14.9%)	128 (14.9%)	
Alcohol intake status				0.1034
No	17,480 (83.6%)	16,779 (83.7%)	701 (81.5%)	
Yes	3,433 (16.4%)	3,274 (16.3%)	159 (18.5%)	
CES-D	11.19 ± 6.62	11.14 ± 6.56	12.31 ± 7.72	<0.001
Total difficulties score				<0.001
No	17,015 (81.4%)	16,371 (81.6%)	644 (74.9%)	
Yes	3,898 (18.6%)	3,682 (18.4%)	216 (25.1%)	
Internalizing problems				0.0285
No	16,538 (79.1%)	15,884 (79.2%)	654 (76%)	
Yes	4,375 (20.9%)	4,169 (20.8%)	206 (24%)	
Externalizing problems				0.1388
No	15,848 (75.8%)	15,215 (75.9%)	633 (73.6%)	
Yes	5,065 (24.2%)	4,838 (24.1%)	227 (26.4%)	
Prosocial behavior				0.0073
No	10,335 (49.4%)	9,871 (49.2%)	464 (54%)	
Yes	10,578 (50.6%)	10,182 (50.8%)	396 (46%)	

Continuous variables presented as Mean ± SD; Categorical variables presented as N (%). SD, standard deviation; N, number of participants.

### Association between preterm birth and psychosocial outcomes

The results from both unadjusted and adjusted logistic regression analyses examining the associations between preterm birth and psychosocial outcomes—including total difficulties, internalizing problems, externalizing problems, and prosocial behavior—are shown in Table 2. Sensitivity analyses using Generalized Estimating Equations (GEE) to account for kindergarten-level clustering yielded nearly identical results (Supplementary Table S1), confirming that the observed associations were not biased by clustering effects. In the non-adjusted model, which adjusts for none, preterm birth is significantly associated with increased odds of total difficulties (OR = 1.49, 95% CI: 1.27–1.75, *p* < 0.001), internalizing problems (OR = 1.20, 95% CI: 1.02–1.41, *p* = 0.0257), and reduced odds of prosocial behavior (OR = 0.83, 95% CI: 0.72–0.95, *p* = 0.0067), with no significant association for externalizing problems (OR = 1.13, 95% CI: 0.97–1.32, *p* = 0.1284). In the fully adjusted model, preterm birth remained significantly associated with increased odds of total difficulties (OR = 1.32, 95% CI: 1.11–1.56, *p* = 0.0015). However, no significant associations were observed for internalizing problems (OR = 1.07, 95% CI: 0.91–1.27, *p* = 0.4153), externalizing problems (OR = 1.02, 95% CI: 0.87–1.20, *p* = 0.8083), or prosocial behavior (OR = 0.89, 95% CI: 0.77–1.02, *p* = 0.1009). Supplementary Table S4 presents the linear regression results for continuous SDQ outcomes (Total difficulties score and Prosocial behavior), which were consistent with the primary findings. Missing data were handled using complete-case analysis in the primary models. A sensitivity analysis employing multiple imputation with 5 imputations yielded generally consistent results for total difficulties and prosocial behavior. However, for internalizing problems, the imputed analysis showed a marginally significant association (OR = 1.17, 95% CI: 1.00–1.36, *p* = 0.0464) that was not observed in the primary complete-case analysis (OR = 1.07, 95% CI: 0.91–1.27, *p* = 0.4153). This discrepancy may reflect differences in missing data patterns between complete cases and imputed datasets. Given the large sample size and minimal missingness in the primary analysis, together with the consistent results from GEE sensitivity analyses, the complete-case findings were considered more reliable. The imputed results should be interpreted with caution.

**Table 2:** Association between preterm and psychosocial outcomes.

Variables	Non-adjusted model	Adjust model
Total difficulties		
Child premature		
Non-preterm	1.0	1.0
Preterm	1.49 (1.27–1.75) < 0.001	1.32 (1.11–1.56) 0.0015
Internalizing problems		
Child premature		
Non-preterm	1.0	1.0
Preterm	1.20 (1.02–1.41) 0.0257	1.07 (0.91–1.27) 0.4153
Externalizing problems		
Child premature		
Non-preterm	1.0	1.0
Preterm	1.13 (0.97–1.32) 0.1284	1.02 (0.87–1.20) 0.8083
Prosocial behavior		
Child premature		
Non-preterm	1.0	1.0
Preterm	0.83 (0.72–0.95) 0.0067	0.89 (0.77–1.02) 0.1009

OR (95%CI) *P* value.

Non-adjusted model adjust for: None.

Adjusted model adjust for: parental gender; parental age; child gender; child age; smoking status; alcohol intake status; education level; annual family income; employment status; marital status; child birth weight; number of children; CES-D.

Primary analyses were based on complete-case data. See Supplementary Table S8 for results after multiple imputation of missing covariates.

### Stratified interaction analyses of the association between preterm birth and psychosocial outcomes

To evaluate potential effect modification by covariates on the associations between preterm birth and psychosocial outcomes, stratified analyses were conducted according to child age and gender, child birth weight, number of children, parental age, parental gender, education level, employment status, marital status, annual family income, smoking status, alcohol intake status, and parental depressive symptoms (CES-D score) (Table 3 and Supplementary Tables S5–S7). After FDR correction, the following interaction effects reached statistical significance. For total difficulties, alcohol intake status significantly modified the association between preterm birth and total difficulties (P for interaction = 0.0017, FDR = 0.0498). For prosocial behavior, parental education level significantly modified the association between preterm birth and prosocial behavior (P for interaction = 0.0012, FDR = 0.0415). With respect to subgroup-specific effects that remained significant after FDR correction, preterm birth was associated with increased odds of total difficulties among the married/cohabiting subgroup (OR = 1.33, FDR = 0.0415) and the annual family income ≥ 100,000 RMB subgroup (OR = 2.03, FDR = 0.0415). Preterm birth was associated with decreased prosocial behavior in the high school diploma/junior college subgroup (OR = 0.70, FDR = 0.0415) and the annual family income ≥ 100,000 RMB subgroup (OR = 0.47, FDR = 0.0023). For internalizing problems and externalizing problems, none of the interaction effects reached statistical significance after FDR correction (all FDR > 0.05). All stratified interaction analyses were exploratory, and significant findings warrant further replication.

**Table 3:** Subgroup analyses of the association between preterm birth and total difficulties.

Variables	Total difficulties	*P*-value	FDR	P interaction	FDR
Child age				0.9022	0.9516
< 4	1.3 (0.91–1.85)	0.1426	0.4826		
≥ 4 to 5	1.34 (1.01–1.77)	0.0407	0.2649		
≥ 5	1.32 (1.02–1.71)	0.038	0.2649		
Child gender				0.5268	0.8758
Boys	1.38 (1.11–1.72)	0.0044	0.0859		
Girls	1.24 (0.96–1.6)	0.1047	0.4187		
Child birth weight (g)				0.3225	0.6921
< 2,500 g or > 4,000 g	1.76 (1.05–2.93)	0.0306	0.2567		
≥2,500 to 4,000 g	1.28 (1.07–1.53)	0.0066	0.102		
Number of children				0.4077	0.8062
1	1.12 (0.82–1.54)	0.4737	0.8523		
2	1.33 (1.07–1.66)	0.0119	0.1308		
≥ 3	1.77 (1.14–2.75)	0.0107	0.126		
Parental age				0.454	0.8456
< 30	1.45 (0.95–2.2)	0.0865	0.3676		
≥ 30 to 45	1.29 (1.07–1.56)	0.0066	0.102		
≥ 45	1.19 (0.4–3.54)	0.7607	0.9101		
Parental gender				0.6581	0.8979
Men	1.24 (0.9–1.72)	0.1933	0.5456		
Female	1.33 (1.1–1.62)	0.004	0.0859		
Education level				0.9447	0.9639
≤ Junior high school	1.42 (1.07–1.89)	0.0153	0.1413		
High school diploma and junior college	1.38 (1.07–1.78)	0.0129	0.1335		
≥ Undergraduate degree	1.09 (0.75–1.57)	0.6474	0.896		
Annual family income, in thousands, ¥				0.6248	0.89
< 30	1.27 (1.01–1.61)	0.0416	0.2649		
≥ 30 to 100	1.15 (0.85–1.56)	0.3499	0.742		
≥ 100	2.03 (1.36–3.04)	<0.001	0.0415		
Employment status					
Working	1.25 (1.02–1.53)	0.0334	0.2649	0.307	0.6767
Not working	1.49 (1.11–2.01)	0.0088	0.1101		
Marital status					
Married/Living with partner	1.33 (1.12–1.58)	0.0011	0.0415	0.6206	0.89
Others	1.13 (0.56–2.29)	0.7294	0.9101		
Smoking status				0.5865	0.8899
No	1.28 (1.07–1.55)	0.0081	0.1101		
Yes	1.47 (0.99–2.19)	0.0561	0.2902		
Alcohol intake status				0.0017	0.0498
No	1.21 (1–1.47)	0.053	0.2847		
Yes	1.76 (1.24–2.5)				
CES-D		0.0534	0.2847	0.7156	0.9101
≥ 16	1.34 (1.1–1.64)	0.004	0.0859		
< 16	1.27 (0.94–1.71)	0.1187	0.4644		

OR (95% CI) *P* value.

Adjusted model adjust for: all other stratification variables except the one being stratified.

FDR, false discovery rate adjusted *p*-value.

Stratification variables were not involved in the adjustment of the respective stratification analysis.

All subgroup analyses were exploratory; FDR correction was applied to control multiple testing bias, significant interaction results need further replication.

## Discussion

In this large-scale cross-sectional study of 20,913 preschool children in Western China, preterm birth was significantly associated with increased total difficulties after full adjustment for child, parental, and household covariates. However, no significant independent associations were observed for internalizing problems, externalizing problems, or prosocial behavior. These findings partially align with the “preterm behavioral phenotype” described in previous literature, which posits that preterm-born children exhibit a characteristic pattern of inattention, anxiety, and social-emotional difficulties rather than pure externalizing problems (26). A longitudinal study of 296 Australian children found that those born moderate-to-late preterm had significant neurodevelopmental challenges at age 9, including lower full-scale IQ and more behavioral difficulties (13). Similarly, a large French cohort reported that 11.1% of very preterm children had abnormal SDQ scores at 5 years, with parent-reported behavioral difficulties highly correlated with school adaptation problems (27). Our study extends these findings to a Chinese preschool population, suggesting that the association between preterm birth and overall psychosocial difficulties may be generalizable across cultural contexts.

The absence of significant associations with internalizing and externalizing problems in our fully adjusted model contrasts with some Western studies. A meta-analysis of attentional deficits following preterm birth confirmed significant impairments in both sustained and selective attention, with greater vulnerability in children born before 32 weeks (28). However, these studies often focused on very or extremely preterm infants, whereas our sample likely comprised predominantly moderate-to-late preterm children given the parent-reported dichotomous classification. The attenuation of associations after covariate adjustment also suggests that family socioeconomic factors and parental mental health may explain a substantial portion of the observed differences between preterm and term-born children in this population.

Several biological and environmental mechanisms have been proposed to potentially contribute to the observed patterns, though these mechanisms cannot be tested with the current cross-sectional design. Biologically, preterm birth may disrupt critical late-gestation processes of cortical maturation, myelination, and neural connectivity, potentially affecting networks involved in attention, emotion regulation, and social cognition (29). Additional hypothesized contributors include neonatal inflammation, oxidative stress, and alterations in the gut–brain axis (30). Environmentally, prolonged NICU stays, altered parent–infant interactions, and cumulative family stressors may interact with biological vulnerabilities, with socioeconomic and parental mental health factors acting as important moderators (31).

Our stratified interaction analyses revealed that parental alcohol intake status modified the association between preterm birth and total difficulties, while parental education level modified the association with prosocial behavior, both surviving FDR correction. These findings are consistent with the family stress model, which posits that socioeconomic disadvantage and parental health behaviors are associated with greater challenges among children facing early biological risk (32). A recent study from the same cohort demonstrated that paternal alcohol consumption was dose-dependently associated with adverse child mental health outcomes, with heavier drinking linked to greater total difficulties (33). The observed interaction between parental education and preterm birth in relation to prosocial behavior is consistent with the “double jeopardy” hypothesis, which predicts that biological and environmental risks show additive associations with child outcomes.

The present study has several methodological limitations. First, the cross-sectional design precludes the determination of causal relationships between preterm birth and psychosocial outcomes, as the temporal sequence and direction of these associations cannot be established. Second, preterm birth status was determined by parental report without medical record verification, and detailed gestational age in weeks was not available. This approach is susceptible to recall bias and potential misclassification, as parents may inaccurately recall gestational age years after delivery. Furthermore, the absence of gestational age data precluded the classification of prematurity severity (extremely, very, moderate-to-late preterm), which have differential neurodevelopmental consequences. The observed associations should therefore be interpreted with caution, and our findings represent associations with broadly defined preterm birth rather than specific subtypes. Third, despite adjusting for a comprehensive set of sociodemographic and behavioral covariates, the possibility of residual or unmeasured confounding persists. Factors not accounted for, such as the severity of neonatal complications (e.g., intraventricular hemorrhage, bronchopulmonary dysplasia) or genetic predispositions, might influence both the risk of preterm birth and child psychosocial development. The absence of these data represents an important limitation. Fourth, the internal consistency of certain SDQ subscales was suboptimal in our sample, with the Cronbach's alpha for the Conduct Problems subscale (0.620) falling slightly below the recommended threshold of 0.70, possibly due to content heterogeneity and floor effects in this community-based sample. Following item-level diagnostic analyses, we removed two items with weak corrected item-total correlations (Items 7 and 23) based on prior psychometric evidence, resulting in acceptable reliability for all revised subscales (*α* ≥ 0.60). However, this modified structure requires cross-validation in independent samples, and the skewed distribution of scores may have attenuated reliability estimates, warranting further investigation in more diverse or clinical populations. Finally, this study was conducted in a single city in Western China, which may limit the generalizability of our findings to other regions or populations. Although stratified cluster sampling was used to enhance representativeness within this urban setting, caution is warranted when extrapolating to rural areas or other geographic regions.

Despite these limitations, the findings carry cautious practical implications for pediatric practice in similar settings. Routine early psychosocial screening using tools such as the SDQ may help identify children with preterm birth history who could benefit from targeted family support or developmental follow-up programs (34). Such strategies should be implemented judiciously to promote equity while avoiding unnecessary labeling, with priority given to integrated early intervention approaches.

## Data Availability

The raw data supporting the conclusions of this article will be made available by the authors, without undue reservation.
