# Supplementary material for: Psychosocial outcomes of preschool children with a history of preterm birth: a cross-sectional observational study
Source: Front Pediatr. 2026 Jul 23;14:1834826. doi: 10.3389/fped.2026.1834826 (PMC13442518; doi:10.3389/fped.2026.1834826)
Supplement: Supplementary file 1 [file Supplementaryfile1.docx]

**Table S1. Sensitivity analysis using Generalized Estimating Equations (GEE) accounting for kindergarten-level clustering, compared with standard logistic regression**

| **Outcome** | **Model** | **GEE OR (95% CI)** | **GEE P-value** | **Logistic OR (95% CI)** | **Logistic P-value** |
| --- | --- | --- | --- | --- | --- |
| Total difficulties | Non-adjusted | 1.49 (1.27-1.75) | <0.001 | 1.49 (1.27-1.75) | <0.001 |
| Total difficulties | Adjusted | 1.32 (1.11-1.57) | 0.002 | 1.32 (1.11-1.56) | 0.002 |
| Internalizing problems | Non-adjusted | 1.2 (1.02-1.41) | 0.025 | 1.2 (1.02-1.41) | 0.026 |
| Internalizing problems | Adjusted | 1.07 (0.91-1.27) | 0.412 | 1.07 (0.91-1.27) | 0.415 |
| Externalizing problems | Non-adjusted | 1.13 (0.97-1.32) | 0.129 | 1.13 (0.97-1.32) | 0.128 |
| Externalizing problems | Adjusted | 1.02 (0.87-1.2) | 0.810 | 1.02 (0.87-1.2) | 0.808 |
| Prosocial behavior | Non-adjusted | 0.83 (0.72-0.95) | 0.007 | 0.83 (0.72-0.95) | 0.007 |
| Prosocial behavior^†^ | Adjusted | 0.89 (0.77-1.02) | 0.101 | 0.89 (0.77-1.02) | 0.101 |
| ^†^Note: GEE models used exchangeable correlation structure with kindergarten as the clustering unit. Adjusted for parental gender, parental age, child gender, child age, smoking status, alcohol intake, education level, annual family income, employment status, marital status, birth weight, number of children, and CES-D. | | | | | |

Abbreviations: GEE, Generalized Estimating Equations; OR, odds ratio; CI, confidence interval.

**Table S2.** **Variance components and intraclass correlation coefficients (ICC) from null models with kindergarten as random intercept**

| **Variables** | **ICC estimate** | **ICC lower** | **ICC upper** |
| --- | --- | --- | --- |
| **Total difficulties** | 0.000493021 | -0.018673795 | 0.019659493 |
| **Internalizing problems** | 0.003263095 | -0.015904539 | 0.02242835 |
| **Externalizing problems** | < 0.001 | -0.01916664 | 0.019166658 |
| **Prosocial behavior** | 0.009862988 | -0.009305411 | 0.029024158 |

**Table S3.** Multicollinearity diagnostics for the regression model of psychosocial outcomes

| **Variables** | **VIF** |
| --- | --- |
| **Child gender** | 1.002298 |
| **Child age** | 1.032588 |
| **Preterm Status** | 1.003564 |
| **Birth weight** | 1.000509 |
| **Parental gender** | 2.397388 |
| **Parental age** | 1.171296 |
| **Education level** | 1.348484 |
| **Employment status** | 1.334453 |
| **Annual family income** | 1.137520 |
| **Number of children** | 1.195307 |
| **Marital status** | 1.029783 |
| **Smoking status** | 2.335482 |
| **Alcohol intake status** | 1.769952 |
| **CES-D** | 1.052386 |

**Table S4.** **Association between preterm and psychosocial outcomes (linear regression)**

| **Exposure** | **Non-adjusted model** | **Adjust model** |
| --- | --- | --- |
| **Total difficulties score** |  |  |
| **Child premature** |  |  |
| **Non-preterm** | Reference | Reference |
| **Preterm** | 1.106 (0.764-1.449) <0.001 | 0.724 (0.408-1.039) <0.001 |
| **Prosocial behavior score** |  |  |
| **Child premature** |  |  |
| **Non-preterm** | Reference | Reference |
| **Preterm** | -0.208 (-0.355--0.062) 0.0052 | -0.107 (-0.249-0.035) 0.1405 |

Data are represented by β (95% CI) P-value.

Non-adjusted model adjust for: None.

Adjusted model adjust for: parental gender; parental age; child gender; child age; smoking status; alcohol intake status; education level; annual family income; employment status; marital status; child birth weight; number of children; CES-D.

**Table S5. Subgroup analyses of the association between preterm birth and internalizing problems**

|  | **Internalizing problems** | **P-value** | **FDR** | **P interaction** | **FDR** |
| --- | --- | --- | --- | --- | --- |
| **Child age** |  |  |  | 0.9104 | 0.9516 |
| < 4 | 1.08 (0.75-1.55) | 0.6884 | 0.9101 |  |  |
| ≥ 4 to 5 | 1.11 (0.84-1.46) | 0.4669 | 0.8523 |  |  |
| ≥ 5 | 1.07 (0.82-1.38) | 0.6229 | 0.89 |  |  |
| **Child gender** |  |  |  | 0.217 | 0.5456 |
| Boys | 1.18 (0.94-1.47) | 0.1504 | 0.4959 |  |  |
| Girls | 0.96 (0.75-1.24) | 0.7589 | 0.9101 |  |  |
| **Child birth weight (g)** |  |  |  | 0.0877 | 0.3676 |
| < 2500 g or > 4000 g | 1.71 (1.03-2.84) | 0.0364 | 0.2649 |  |  |
| ≥2500 to 4000 g | 1.02 (0.86-1.22) | 0.7868 | 0.9171 |  |  |
| **Number of children** |  |  |  | 0.2989 | 0.6745 |
| 1 | 1.19 (0.88-1.61) | 0.2642 | 0.62 |  |  |
| 2 | 0.97 (0.77-1.21) | 0.7612 | 0.9101 |  |  |
| ≥ 3 | 1.36 (0.88-2.11) | 0.1688 | 0.5123 |  |  |
| **Parental age** |  |  |  | 0.0502 | 0.2847 |
| < 30 | 1.51 (1-2.28) | 0.0518 | 0.2847 |  |  |
| ≥ 30 to 45 | 1.01 (0.83-1.21) | 0.9475 | 0.9639 |  |  |
| ≥ 45 | 1.21 (0.4-3.65) | 0.7322 | 0.9101 |  |  |
| **Parental gender** |  |  |  | 0.6337 | 0.89 |
| Men | 1.01 (0.73-1.4) | 0.9637 | 0.9748 |  |  |
| Female | 1.1 (0.9-1.33) | 0.3597 | 0.7503 |  |  |
| **Education level** |  |  |  | 0.8377 | 0.9512 |
| ≤ Junior high school | 1.24 (0.94-1.65) | 0.1326 | 0.4797 |  |  |
| High school diploma and junior college | 1.17 (0.91-1.5) | 0.2315 | 0.558 |  |  |
| ≥ Undergraduate degree | 0.74 (0.5-1.1) | 0.1336 | 0.4797 |  |  |
| **Annual family income, in thousands, ¥** |  |  |  | 0.224 | 0.5475 |
| < 30 | 1.15 (0.91-1.44) | 0.2464 | 0.5861 |  |  |
| ≥ 30 to 100 | 0.92 (0.68-1.24) | 0.5852 | 0.8899 |  |  |
| ≥ 100 | 1.34 (0.87-2.04) | 0.181 | 0.5288 |  |  |
| **Employment status** |  |  |  | 0.5074 | 0.8758 |
| Working | 1.04 (0.85-1.27) | 0.7133 | 0.9101 |  |  |
| Not working | 1.17 (0.86-1.57) | 0.3147 | 0.6838 |  |  |
| **Marital status** |  |  |  | 0.5304 | 0.8758 |
| Married/Living with partner | 1.06 (0.89-1.26) | 0.4871 | 0.8574 |  |  |
| Others | 1.25 (0.61-2.52) | 0.5428 | 0.8758 |  |  |
| **Smoking status** |  |  |  | 0.3666 | 0.7503 |
| No | 1.03 (0.86-1.24) | 0.7378 | 0.9101 |  |  |
| Yes | 1.31 (0.88-1.93) | 0.1833 | 0.5288 |  |  |
| **Alcohol intake status** |  |  |  |  |  |
| No | 1.01 (0.84-1.22) | 0.9137 | 0.9516 | 0.1562 | 0.4959 |
| Yes | 1.31 (0.92-1.88) | 0.1331 | 0.4797 | 0.1227 | 0.4694 |
| **CES-D** |  |  |  |  |  |
| ≥ 16 | 0.98 (0.8-1.21) | 0.8638 | 0.9516 |  |  |
| < 16 | 1.3 (0.97-1.75) | 0.0842 | 0.3676 |  |  |

Note: OR (95% CI) P value

Adjusted model adjust for: all other stratification variables except the one being stratified

FDR = False Discovery Rate adjusted p-value.

Stratification variables were not involved in the adjustment of the respective stratification analysis.

All subgroup analyses were exploratory; FDR correction was applied to control multiple testing bias, significant interaction results need further replication.

**Table S6. Subgroup analyses of the association between preterm birth and externalizing problems**

|  | **Externalizing problems** | **P-value** | **FDR** | **P interaction** | **FDR** |
| --- | --- | --- | --- | --- | --- |
| **Child age** |  |  |  | 0.214 | 0.5456 |
| < 4 | 0.89 (0.64-1.25) | 0.5119 | 0.8758 |  |  |
| ≥ 4 to 5 | 1.18 (0.91-1.53) | 0.211 | 0.5456 |  |  |
| ≥ 5 | 0.99 (0.77-1.27) | 0.9106 | 0.9516 |  |  |
| **Child gender** |  |  |  | 0.5388 | 0.8758 |
| Boys | 1.08 (0.87-1.33) | 0.4798 | 0.853 |  |  |
| Girls | 0.96 (0.75-1.23) | 0.7683 | 0.9101 |  |  |
| **Child birth weight (g)** |  |  |  | 0.6128 | 0.89 |
| < 2500 g or > 4000 g | 0.92 (0.55-1.56) | 0.7671 | 0.9101 |  |  |
| ≥2500 to 4000 g | 1.04 (0.88-1.23) | 0.6256 | 0.89 |  |  |
| **Number of children** |  |  |  | 0.679 | 0.9101 |
| 1 | 0.95 (0.71-1.27) | 0.7192 | 0.9101 |  |  |
| 2 | 1.02 (0.83-1.26) | 0.8498 | 0.9516 |  |  |
| ≥ 3 | 1.32 (0.85-2.04) | 0.2148 | 0.5456 |  |  |
| **Parental age** |  |  |  | 0.0239 | 0.21 |
| < 30 | 1.51 (1.02-2.24) | 0.0395 | 0.2649 |  |  |
| ≥ 30 to 45 | 0.96 (0.8-1.15) | 0.6517 | 0.896 |  |  |
| ≥ 45 | 0.96 (0.36-2.55) | 0.9427 | 0.9639 |  |  |
| **Parental gender** |  |  |  | 0.5546 | 0.8758 |
| Men | 0.94 (0.68-1.29) | 0.6884 | 0.9101 |  |  |
| Female | 1.06 (0.88-1.27) | 0.5573 | 0.8758 |  |  |
| **Education level** |  |  |  | 0.6106 | 0.89 |
| ≤ Junior high school | 1.11 (0.83-1.48) | 0.4746 | 0.8523 |  |  |
| High school diploma and junior college | 1 (0.78-1.27) | 0.9773 | 0.9829 |  |  |
| ≥ Undergraduate degree | 1 (0.73-1.35) | 0.9832 | 0.9832 |  |  |
| **Annual family income, in thousands, ¥** |  |  |  | 0.2144 | 0.5456 |
| < 30 | 1.04 (0.83-1.31) | 0.7082 | 0.9101 |  |  |
| ≥ 30 to 100 | 0.81 (0.6-1.09) | 0.1578 | 0.4959 |  |  |
| ≥ 100 | 1.64 (1.14-2.34) | 0.007 | 0.102 |  |  |
| **Employment status** |  |  |  | 0.844 | 0.9516 |
| Working | 1.02 (0.85-1.23) | 0.8136 | 0.9359 |  |  |
| Not working | 1.07 (0.8-1.45) | 0.6369 | 0.89 |  |  |
| **Marital status** |  |  |  | 0.5262 | 0.8758 |
| Married/Living with partner | 1.04 (0.89-1.23) | 0.6193 | 0.89 |  |  |
| Others | 0.8 (0.39-1.66) | 0.5562 | 0.8758 |  |  |
| **Smoking status** |  |  |  | 0.6905 | 0.9101 |
| No | 1.01 (0.85-1.2) | 0.9025 | 0.9516 |  |  |
| Yes | 1.12 (0.76-1.66) | 0.5717 | 0.8893 |  |  |
| **Alcohol intake status** |  |  |  | 0.137 | 0.4822 |
| No | 0.97 (0.81-1.17) | 0.7755 | 0.9101 |  |  |
| Yes | 1.25 (0.88-1.77) | 0.2067 | 0.5456 |  |  |
| **CES-D** |  |  |  | 0.8877 | 0.9516 |
| ≥ 16 | 1.03 (0.86-1.25) | 0.7201 | 0.9101 |  |  |
| < 16 | 1.03 (0.76-1.39) | 0.8631 | 0.9516 |  |  |

Note: OR (95% CI) P value

Adjusted model adjust for: all other stratification variables except the one being stratified

FDR = False Discovery Rate adjusted p-value.

Stratification variables were not involved in the adjustment of the respective stratification analysis.

All subgroup analyses were exploratory; FDR correction was applied to control multiple testing bias, significant interaction results need further replication.

**Table S7. Subgroup analyses of the association between preterm birth and prosocial behavior**

|  | **Prosocial behavior** | **P-value** | **FDR** | **P interaction** | **FDR** |
| --- | --- | --- | --- | --- | --- |
| **Child age** |  |  |  | 0.9104 | 0.9516 |
| < 4 | 0.87 (0.65-1.18) | 0.3763 | 0.7598 |  |  |
| ≥ 4 to 5 | 0.84 (0.67-1.06) | 0.141 | 0.4826 |  |  |
| ≥ 5 | 0.92 (0.74-1.14) | 0.4286 | 0.8289 |  |  |
| **Child gender** |  |  |  | 0.4131 | 0.8078 |
| Boys | 0.84 (0.7-1.02) | 0.0727 | 0.3281 |  |  |
| Girls | 0.92 (0.75-1.14) | 0.4564 | 0.8456 |  |  |
| **Child birth weight (g)** |  |  |  | 0.7505 | 0.9101 |
| < 2500 g or > 4000 g | 0.97 (0.61-1.52) | 0.8814 | 0.9516 |  |  |
| ≥2500 to 4000 g | 0.87 (0.75-1) | 0.0579 | 0.2911 |  |  |
| **Number of children** |  |  |  | 0.7757 | 0.9101 |
| 1 | 0.92 (0.71-1.19) | 0.5315 | 0.8758 |  |  |
| 2 | 0.88 (0.73-1.05) | 0.1563 | 0.4959 |  |  |
| ≥ 3 | 0.8 (0.55-1.19) | 0.2722 | 0.6304 |  |  |
| **Parental age** |  |  |  | 0.8193 | 0.9363 |
| < 30 | 0.94 (0.65-1.38) | 0.7624 | 0.9101 |  |  |
| ≥ 30 to 45 | 0.88 (0.75-1.02) | 0.0898 | 0.3677 |  |  |
| ≥ 45 | 0.6 (0.27-1.3) | 0.1966 | 0.5456 |  |  |
| **Parental gender** |  |  |  |  |  |
| Men |  |  |  | 0.551 | 0.8758 |
| Female | 0.81 (0.61-1.09) | 0.162 | 0.5003 |  |  |
| **Education level** | 0.9 (0.76-1.05) | 0.1743 | 0.5201 | 0.0012 | **0.0415** |
| ≤ Junior high school | 1.18 (0.91-1.52) | 0.2204 | 0.5463 |  |  |
| High school diploma and junior college | 0.7 (0.56-0.87) | 0.0011 | **0.0415** |  |  |
| ≥ Undergraduate degree | 0.9 (0.69-1.18) | 0.446 | 0.8448 |  |  |
| **Annual family income, in thousands, ¥** |  |  |  | 0.8979 | 0.9516 |
| < 30 | 1.01 (0.82-1.24) | 0.92 | 0.9525 |  |  |
| ≥ 30 to 100 | 0.99 (0.78-1.24) | 0.9137 | 0.9516 |  |  |
| ≥ 100 | 0.47 (0.34-0.66) | <0.001 | **0.0023** |  |  |
| **Employment status** |  |  |  | 0.3657 | 0.7503 |
| Working | 0.84 (0.72-0.99) | 0.0421 | 0.2649 |  |  |
| Not working | 0.97 (0.74-1.26) | 0.795 | 0.9205 |  |  |
| **Marital status** |  |  |  | 0.3799 | 0.7598 |
| Married/Living with partner | 0.86 (0.75-1) | 0.0448 | 0.272 |  |  |
| Others | 1.2 (0.63-2.3) | 0.576 | 0.8893 |  |  |
| **Smoking status** |  |  |  | 0.0719 | 0.3281 |
| No | 0.92 (0.79-1.07) | 0.2911 | 0.6654 |  |  |
| Yes | 0.62 (0.42-0.91) | 0.0147 | 0.1413 |  |  |
| **Alcohol intake status** |  |  |  | 0.3076 | 0.6767 |
| No | 0.91 (0.78-1.06) | 0.2096 | 0.5456 |  |  |
| Yes | 0.73 (0.52-1.02) | 0.0612 | 0.2929 |  |  |
| **CES-D** |  |  |  | 0.6371 | 0.89 |
| ≥ 16 | 0.86 (0.74-1.01) | 0.0616 | 0.2929 |  |  |
| < 16 | 0.89 (0.65-1.21) | 0.4464 | 0.8448 |  |  |

Note: OR (95% CI) P value

Adjusted model adjust for: all other stratification variables except the one being stratified

FDR = False Discovery Rate adjusted p-value.

Stratification variables were not involved in the adjustment of the respective stratification analysis.

All subgroup analyses were exploratory; FDR correction was applied to control multiple testing bias, significant interaction results need further replication.

**Table S8. Associations between preterm birth and psychosocial outcomes in preschool children after imputing the baseline missing values**

| Exposure | Non-adjusted model | Adjust model |
| --- | --- | --- |
| Total difficulties |  |  |
| **Child premature** |  |  |
| Non-preterm | 1.0 | 1.0 |
| Preterm | 1.54 (1.34-1.77) < 0.001 | 1.37 (1.17-1.59) < 0.001 |
| **Internalizing problems** |  |  |
| **Child premature** |  |  |
| Non-preterm | 1.0 | 1.0 |
| Preterm | 1.3(1.13-1.49 ) < 0.001 | 1.17(1-1.36) 0.0464 |
| **Externalizing problems** |  |  |
| **Child premature** |  |  |
| Non-preterm | 1.0 | 1.0 |
| Preterm | 1.23 (1.07- 1.4) 0.0027 | 1.13(0.98-1.31) 0.0923 |
| **Prosocial behavior** |  |  |
| **Child premature** |  |  |
| Non-preterm | 1.0 | 1.0 |
| Preterm | 0.85 (0.76-0.96)0.0111 | 0.92 (0.8-1.04) 0.1883 |

Note: OR (95%CI) P value.

Non-adjusted model adjust for: None.

Adjusted model adjust for: parental gender; parental age; child gender; child age; smoking status; alcohol intake status; education level; annual family income; employment status; marital status; child birth weight; number of children; CES-D.
